# Modulating break types induces divergent low band EEG processes during post-break improvement: A power spectral analysis

**DOI:** 10.3389/fnhum.2022.960286

**Published:** 2022-09-12

**Authors:** Sujie Wang, Li Zhu, Lingyun Gao, Jingjia Yuan, Gang Li, Yu Sun, Peng Qi

**Affiliations:** ^1^Key Laboratory for Biomedical Engineering of Ministry of Education, Department of Biomedical Engineering, Zhejiang University, Hangzhou, China; ^2^School of Physical Education and Health Science, Guangxi University for Nationalities, Nanning, China; ^3^College of Mathematical Medicine, Zhejiang Normal University, Jinhua, China; ^4^Department of Neurology, Sir Run Run Shaw Hospital, Zhejiang University School of Medicine, Hangzhou, China; ^5^Department of Control Science and Engineering, College of Electronics and Information Engineering, Tongji University, Shanghai, China

**Keywords:** mental fatigue recovery, time-on-task (TOT), sustained attention, EEG, power spectral density, exercise

## Abstract

Conventional wisdom suggests mid-task rest as a potential approach to relieve the time-on-task (TOT) effect while accumulating evidence indicated that acute exercise might also effectively restore mental fatigue. However, few studies have explored the neural mechanism underlying these different break types, and the results were scattered. This study provided one of the first looks at how different types of fatigue-recovery break exerted influence on the cognitive processes by evaluating the corresponding behavioral improvement and neural response (EEG power spectral) in a sustained attention task. Specifically, 19 participants performed three sessions of psychomotor vigilance tasks (PVT), with one session including a continuous 30-min PVT while the other two sessions additionally inserted a 15-min mid-task cycling and rest break, respectively. For behavioral performance, both types of break could restore objective vigilance transiently, while subjective feeling was only maintained after mid-task rest. Moreover, divergent patterns of EEG change were observed during post-break improvement. In detail, relative theta decreased and delta increased immediately after mid-task exercise, while decreased delta was found near the end of the rest-inserted task. Meanwhile, theta and delta could serve as neurological indicators to predict the reaction time change for exercise and rest intervention, respectively. In sum, our findings provided novel evidence to demonstrate divergent neural patterns following the mid-task exercise and rest intervention to counter TOT effects, which might lead to new insights into the nascent field of neuroergonomics for mental fatigue restoration.

## Introduction

In contemporary society, sustaining steady attention for a long period is a challenging yet necessary cognitive process on numerous occasions (Thomson et al., 2015; Esterman and Rothlein, 2019). However, prolonged mental activities are inevitably accompanied by impaired executive control ability (Langner and Eickhoff, 2013; Thomson et al., 2015) and deteriorated performance in attention-related tasks (Boksem et al., 2006; Wascher et al., 2014), like an increased propensity for lapses/errors and slowed reaction time. Collectively, these objective declines are known as the effects of time-on-task (TOT) (Mackworth, 1948; Lim et al., 2010, 2013). TOT-altered effects have been repeatedly revealed in on-the-job lapses in industries where workers are required to work for long periods without rest, leading to lowered productivity and increased safety risks. Because of these undesirable yet preventable consequences, emerging efforts have been made to find an efficient countermeasure for the TOT effect (Helton and Russell, 2017).

As a non-invasive measurement, EEG has been widely employed in mental fatigue studies and provided several promising results (Borghini et al., 2014; Thomson et al., 2015; Helfrich and Knight, 2016; Sun et al., 2017; Gao et al., 2019). For instance, accumulating evidence suggested that increased low-band (i.e., 1–7 Hz) neural activity (Craig et al., 2012) was broadly recognized as an indication of increasing mental fatigue. In a recent meta-analysis, increased theta band (i.e., 4–7 Hz) neural activity was reported to serve as a sailent neural biomarker of mental fatigue across the frontal, central, and posterior cortical sites (Tran et al., 2020). Besides, the increase of delta (i.e., 1–4 Hz) was also repeatedly observed during mental fatigue in aircraft pilots and car drivers (Borghini et al., 2014), visual attention tasks (Ko et al., 2017), and steady-state visual evoked potential (SSVEP) experiments (Cao et al., 2014). Based on these fatigue-induced EEG findings, Clayton et al. proposed the oscillation model of sustained attention, where they postulated that frontal theta was associated with monitoring and control of cognitive processes, and increased frontomedial-theta power might manifest detection of a mismatch between current and desired levels of attention (Clayton et al., 2015). In sum, a relatively comprehensive picture pertaining to the neural characteristics of mental fatigue has been obtained. Nonetheless, only until recently, studies started to quantitatively investigate the biobehavioral performance of break-related fatigue recovery and reported mixed findings (Wascher et al., 2014; Lim et al., 2016).

Conventional wisdom suggests that mid-task rest is a potential intervention to reduce mental fatigue and burst cognitive performance. For example, Lim investigated the effect of different rest durations (i.e., 1, 5, and 10 min) on mental fatigue recovery and indicated that longer breaks were associated with better restoration of behavioral performance (Lim et al., 2016). Moreover, Helton et al. showed that during the vigilance task, participants performed better after resting than switching to a secondary task (Helton and Russell, 2015). In addition to the well-known rest intervention approach, accumulating meta-analytical evidence (Chang et al., 2012) and reviews (Pontifex et al., 2019; Tomporowski and Pesce, 2019) indicated that acute moderate-intensity exercise was effective for improving cognitive performance, especially in restoring attention (Fernandes et al., 2019). An increased physical activity level was also found to be associated with reduced subjective fatigue (Southard et al., 2015; Sonnentag, 2018). However, in comparison with those consistent findings indicating that breaks restore fatigue-related behavioral assessments, the results about their underlying neural mechanism are relatively mixed. For instance, Phipps-Nelson et al. showed that rest break did improve driving performance after fatigue, yet failed to identify any beneficial effect of rest on EEG characteristics (Phipps-Nelson et al., 2011). Besides, Lim et al. did not observe any time by condition interaction of EEG activities between successive and rest-inserted auditory oddball tasks (Lim et al., 2013). Moreover, in our recent work investigating the neural mechanism of post-exercise performance restoration, no significant difference was observed between successive task and exercise-inserted task in static dynamic functional connectivity (FC) analysis (Gao et al., 2020), and similar dynamic FC development trends were exhibited between tasks with exercise-break and rest-break (Gao et al., 2022). Generally, these scattered studies still suggested a nascent trend in the field of neuroergonomics to further uncover the divergent underlying neural mechanisms of different fatigue recovery approaches.

This research gap has been a key motivation for this study. In particular, our study investigated this undiscovered post-intervention improvement difference by comparing the neural activity change and cognitive pattern of mid-task rest and exercise. In the laboratory, tests of sustained attention have been particularly amenable for inducing the TOT effect because of their reliability and validity. Therefore, a previously validated psychomotor vigilance task (PVT) paradigm was used in this work. Specifically, a within-subject three-session design was adopted, with one session consisting of a continuous 30-min PVT while the other two sessions additionally included a 15-min mid-task rest break and a 15-min cycling break, respectively. Unlike our previous research that focused on network analysis and revealed non-significant effects (Gao et al., 2020), EEG power spectral analysis was utilized to interrogate the processes in a low-frequency band in this study, which aimed to explore how different types of fatigue-recovery break exerted influence on the cognitive processes from a new perspective. In detail, delta (1–4 Hz) and theta (4–7Hz) bands were proved to be sensitive to wakeful and sleep mental states and therefore considered a potential biomarker for the wakefulness assessment (Tran et al., 2020). We further examined the association between these brain activities and vigilance behavioral performance. Based on the reported beneficial effects of exercise (Pontifex et al., 2019) and rest (Lim et al., 2016), we hypothesized that both interventions would relieve TOT effects from a biobehavioral perspective. Based on the divergent characteristics of the two types of intervention, we further hypothesized that exercise break and rest break exerted different impacts on cognitive processes of sustained attention, which might be manifested as different spatio-spectral power activity changes.

## Methods and materials

### Participants

Twenty-one healthy young adults from Zhejiang University were recruited in this study *via* an online advertisement. Among them, two subjects were excluded due to data acquisition issues, and the remaining 19 subjects (male/female = 13/6, age = 22.16 ± 0.65 years) were included for the following analysis. All participants had normal or corrected-to-normal vision. None of them reported cardiovascular, cognitive disorder, color blindness, or any acute illness that might influence the cognitive task performance. Each participant was informed of the experimental protocol and potential risks through a telephone interview. They were required to get a full night of sleep (> 7 h, recorded by HUAWEI-B19 bracelet) for two continuous nights and were instructed to abstain from caffeine and alcohol and avoid vigorous physical activity for 7 h before the experiment. Those participants who failed to meet the requirements were either ruled out from the experiment or rescheduled. Upon arriving at the lab, written informed consent and Physical Activity Readiness Scale (PARS) were provided to exclude any contraindication during exercise. This study was approved by the Institutional Review Board of Zhejiang University and was carried out in compliance with the Declaration of Helsinki.

### Experimental protocol

**Figure 1:**
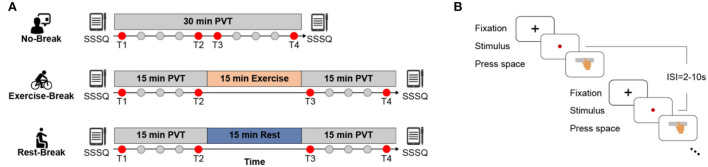
Schematic illustration of the experimental protocol. **(A)** No-Break session includes a 30-min continuous PVT task, while Exercise-Break and Rest-Break session are intervened by a 15 min mid-task exercise and mid-task rest, respectively. Of note, T1 and T4 that correspond to the first and last 3 min of each session; and T2 and T3 that represent the pre-break and post-break 3 min of each session were defined to investigate the immediate effect (T2 vs. T3) and general effect (T1 vs. T4) for post-break intervention. **(B)** The detailed paradigm of PVT task. Subjects were required to press the space bar as quickly as possible after monitoring the stimulus (red dot), with 2–10 s inter-stimulus-intervals (ISI).

A within-subject design was adopted in this work to explore the effects of receiving different types of breaks on mental fatigue recovery, where each participant was requested to finish three sessions in a counterbalanced manner with approximate 1-week interval (Figure 1). During each session (namely, *No-Break, Exercise-Break*, and *Rest-Break* session, respectively), participants were required to perform a 30-min PVT with or without a break. The design of PVT was the same as in our previous studies (Gao et al., 2020). Briefly, during the test, subjects were required to monitor a red dot that appeared on the screen and responded to this stimulus by pressing the space bar as soon as possible. Intervals between trials varied randomly from 2 to 10 s (mean = 6 s).

In the *No-Break* session, participants were required to perform the PVT task continuously without a break, while during the *Rest-* or *Exercise-Break* session, participants were required to perform PVT tasks with a 15-min still sitting with eyes fixed on a black cross (namely *mid-task rest)* or cycling on a Monark 975 stationary exercise bicycle (namely *mid-task exercise*) in the middle of the tasks. Sessions were arranged at the same time of the day to eliminate the impact of circadian rhythm (Pontifex et al., 2019). The *mid-task rest* was introduced with the notion that rest breaks induced mental fatigue recovery (Lim et al., 2013; Sun et al., 2017; Qi et al., 2020), while the *mid-task exercise* corresponded to the view that the moderate-intensity exercise led to cognitive improvement (Chang et al., 2012). The intensity of the cycling break was estimated at individual-level using the following formula:


$$\begin{array}{l} \text{Target HR}=\left(HR_{max}-HR_{rest}\right)\;\times \;55\sim 65\%+HR_{rest} \end{array}$$

where HR stands for heart rate, and *HR*_*max*_ was set as 220 – age, *HR*_*rest*_ was obtained after participants resting for 5~7 min (recorded by the Polar Vantage V). In addition, subjective feelings of mental states were assessed *via* the Short Stress State Questionnaire (SSSQ) (Helton et al., 2005; Lim et al., 2016; Qi et al., 2020) before and immediately after each session. Three minutes was determined as the length of an epoch to better illustrate the transient effect of post-break while including enough PVT trials for a stable behavioral and electrophysiological metrics estimation.

### EEG recording and pre-processing

High-density continuous EEG was recorded from 64 Ag/AgCl scalp electrodes (model: Brain Products MR Plus, Germany) according to the International 10–20 system during the 30-min PVT for three sessions. The sample rate was set at 2,000 Hz with reference to the FCz. The impedance was kept below 5 kΩ during the whole data collection. In the offline analysis, a previously validated standard EEG preprocessing pipeline was adopted. Briefly, all data were down-sampled to 256 Hz, bandpass filtered into 1–40 Hz, 50 Hz notch filtered, and average re-referenced. Independent component analysis (ICA) (Jung et al., 1997) was further used to detect and eliminate artifacts (i.e., EOG and eye movements). Then for each 3-min epoch, the obtained EEG data were de-trended and segmented into 6-s trials (corresponding to the average time of one behavioral trial in the PVT). EEG data in an epoch with a voltage >100 uV was rejected (Zhao et al., 2012). For all preprocessing steps, customized codes and the EEGLab toolbox (Delorme and Makeig, 2004) were used in MATLAB 2020B.

### Power analysis

Each 6-s EEG data trial was divided into segments using a 100% Hamming window of 1 s, then the power spectral density (PSD) was estimated by Welch's method, and specially focused on two low-frequency bands: delta band (1–4 Hz) and theta band (4–7 Hz) for their potential sensitivity toward mental states (Tran et al., 2020). For each channel, relative power (*P*_δ_ and *P*_θ_) was estimated as the ratio of the specific band power to the total power spectral that was estimated with a frequency range of 1–40 Hz. For each 3-min epoch, averaged relative power was obtained from all included trials.

**Figure 2:**
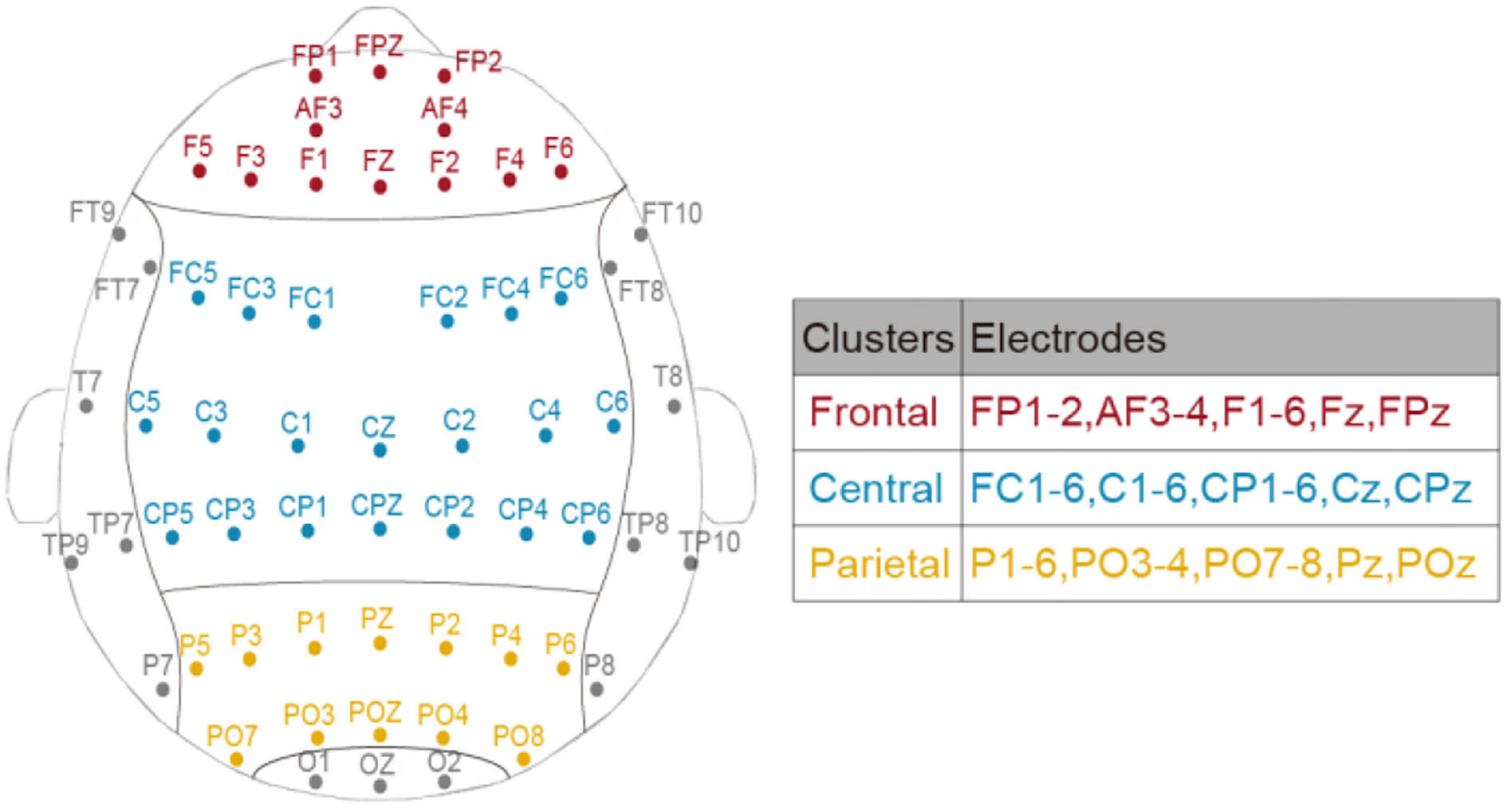
The assignment of electrodes in frontal, central, and parietal clusters.

Meta-analysis suggested that spectral EEG across frontal, central, and posterior cortical regions was related to mental fatigue, and the effect size was large for the central region and moderate for the frontal and posterior regions (Tran et al., 2020). Considering these region-dependent alterations of EEG power during TOT (Craig et al., 2012; Lim et al., 2013), three bilateral clusters (corresponding to frontal, central, and parietal areas) were divided to better investigate the restorative effect of different breaks. The assignment of electrodes in each cluster is displayed in Figure 2.

### Statistical analysis

#### Behavioral variables

Consistent with the previous study (Gao et al., 2022), we primarily focused on the reaction time (RT) for the analysis of behavioral performance, as it was directly affected by fatigue (Dorrian et al., 2004). In detail, an RT longer than 500 ms was considered a lapse, and a RT shorter than 100 ms was considered a false alarm. Hence, only RTs between 100 and 500 ms after the stimulus onset were included in the following statistical analyses. To investigate the effectiveness of the PVT-induced TOT effect, one-way repeated-measures ANOVA for RT in ten 3-min bins was conducted in the *No-Break* session, as well as an assessment of the slope of RT. For the effect of mid-task breaks, previous evidence showed that post-break behavioral improvements were transient (Lim and Kwok, 2015; Lim et al., 2016). Therefore, we evaluated the intervention effect of breaks from two aspects: immediate and general effect. Of note, T1 and T4 correspond to the first and last 3 min of each session; and T2 and T3 represent the pre-break and post-break 3 min of each session and were defined to investigate the immediate effect (T2 vs. T3) and general effect (T1 vs. T4) for post-break intervention. Specifically, a two (Time: T2 and T3) by three (Session: *No-Break, Exercise-Break*, and *Rest-Break*) repeated measures two-way ANOVA on RT was adopted to explore the immediate influence of breaks, while another repeated measures two-way ANOVA with Time (T1 and T4) by Session (*No-Break, Exercise-Break*, and *Rest-Break*) on RT was conducted to investigate the long-lasting general effect.

#### Subjective feelings

As mentioned previously, the subjective feelings of participants were also assessed *via* SSSQ prior to and immediately after the PVT. Heuristically, the 24-item SSSQ could be divided into three categories: engagement, worry, and distress (Helton et al., 2005). To assess the subjective mental states before and after the tasks, a separate two (Time: Pre-session, Post-session) by three (Session: *No-Break, Exercise-Break*, and *Rest-Break*) repeated measures ANOVA was conducted on the SSSQ scores of each category.

#### EEG power

To comprehensively depict the break-related spectral changes in the low-frequency range, averaged EEG power ratio of two frequency bands (delta and theta) in the three clusters (Figure 2) was obtained. Similar to the behavioral variable, the immediate effect (i.e., T2 vs. T3) and general effect (i.e., T1 vs. T4) of the mid-task breaks were assessed through separate two by three repeated measures two-way ANOVA with Time by Session (*No-Break, Exercise-Break*, and *Rest-Break*). Specifically, the dependent variables were the multiband EEG power ratio in different brain clusters.

#### Correlation

For the frequency band displaying the significant spectral difference in the last step, the correlation analysis was conducted between the changes of RT (ΔRT, in percentage) and relative EEG (*P*_δ_, Δ*P*_δ_, *P*_θ_, Δ*P*_θ_) to investigate the behavior–EEG relationship in the immediate effects and general effects in different sessions.

Normality and sphericity of data were checked for statistical analysis. A value of *p* < 0.05 was considered significant. All statistical processes were performed using the SPSS 25.0 software package (SPSS, Chicago, IL).

## Results

### Behavioral results

**Figure 3:**
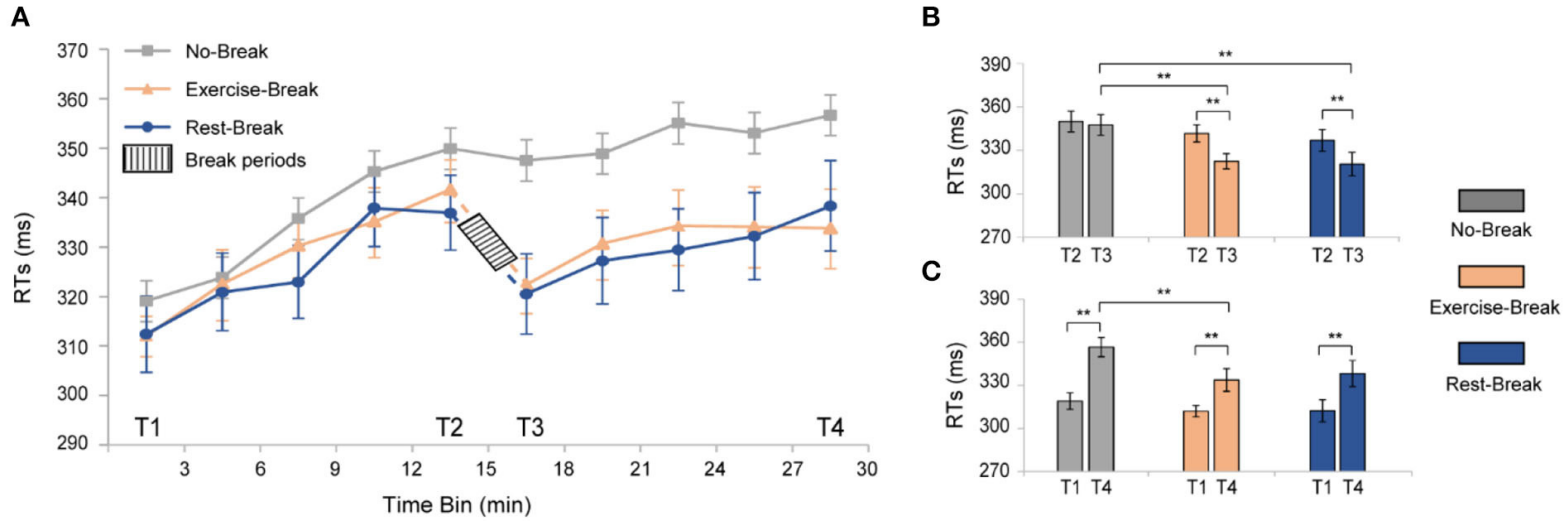
Behavioral results for the three different sessions. **(A)** RTs of PVT task segmented into 3-min bin. **(B)** *post-hoc* analyses for the immediate effect of mid-task interventions. **(C)** *Post-hoc* analyses for the general effect of the mid-task breaks. **p < 0.01. Error bars represent the mean ± SEM.

To investigate the time-on-task (TOT) effect induced by PVT, we averaged the reaction time (RT) in the *No-Break* session into 10 3-min bins to evaluate the trend of behavioral performance over the task (Figure 3A, gray curve). As expected, performing PVT led to a significant TOT effect, manifesting increments in RT similar to our previous studies (Sun et al., 2017). Specifically, a significant increase [*F*_(3, 54)_ = 22.270, *p* < 0.001, η^2^ = 0.553] of reaction time was observed from T1 to T4. The individually fitted linear slope of the RT also showed a performance decline throughout the task (slope Mean ± SD = 0.022 ± 0.004), in which averaged RT was 11.78% longer during T4 (most fatigued) in comparison with T1 (most vigilant).

For the immediate effect (T2 vs. T3) of mid-task breaks (Figure 3B), there were significant main effects for time-by-session interaction [*F*_(2, 36)_ = 4.942, *p* = 0.013, η^2^ = 0.215], time [*F*_(1, 18)_ = 25.713, *p* < 0.001, η^2^ = 0.588], and session [*F*_(2, 36)_ = 7.421, *p* = 0.002, η^2^ = 0.292]. Following *post-hoc* analyses indicated that the significant interaction effect was attributed to the decrease of RT in both the *Exercise-Break* session [*F*(1, 18) = 16.448, *p* = 0.001, η^2^ = 0.477] and the *Rest-Break* session [*F*_(1, 18)_ = 17.838, *p* = 0.001, η^2^ = 0.498]. However, no significant difference was observed between the *Exercise-Break* session and *Rest-Break* session in their post-break RT, indicating a similar vigilance improvement immediately after different interventions.

The general effect (T1 vs. T4) of mid-task breaks was also tested (Figure 3C). Significant main effects were found for time [*F*_(1, 18)_ = 65.451, *p* < 0.001, η^2^ = 0.784] and sessions [*F*_(2, 36)_ = 4.655, *p* = 0.016, η^2^ = 0.205], but no time-by-session interaction [*F*_(2, 36)_ = 2.059, *p* = 0.142, η^2^ = 0.103]. Follow-up *post-hoc* comparisons for the main time effect suggested that RT in T4 was significantly longer than that in T1 (*p* < 0.001 in all sessions). Specifically, compared with T1, RTs in *Exercise-Break* and *Rest-Break* sessions increased by 6.970 and 8.297%, respectively. In addition, *post-hoc* comparisons for the main session effect indicated that in the T4, the RT in the *Exercise-Break* session was significantly shorter than that in the *No-Break* session [*F*_(1, 18)_ = 8.431, *p* = 0.009, η^2^ = 0.319].

In terms of subjective states, significant main effect of time was found on both engagement [*F*_(1, 18)_ = 21.535, *p* < 0.001, η^2^ = 0.545] and distress [*F*_(1, 18)_ = 8.568, *p* = 0.009, η^2^ = 0.322] scores, but no significant difference of session [engagement: *F*_(2, 36)_ = 1.032, *p* = 0.367, η^2^ = 0.054; distress: *F*_(2, 36)_ = 1.762, *p* = 0.186, η^2^ = 0.089] and time-by-session [engagement: *F*_(2, 36)_ = 0.804, *p* = 0.455, η^2^ = 0.043; distress: *F*_(2, 36)_ = 0.113, *p* = 0.894, η^2^ = 0.006] interaction. Further inspection of the main effect in time suggested that for *No-Break* and *Exercise-Break* session, participants showed significantly increased distress [*No-Break* session: *F*_(1, 18)_ = 13.986, *p* = 0.001, η^2^ = 0.437; *Exercise-Break* session: *F*_(1, 18)_ = 6.495, *p* = 0.02, η^2^ = 0.265] as well as less engagement [*No-Break* session: *F*_(1, 18)_ = 17.618, *p* = 0.001, η^2^ = 0.495; *Exercise-Break* session: *F*_(1, 18)_ = 4.015, *p* = 0.06, η^2^ = 0.182] in the post-session compared with pre-session, indicating a similar trend of subjective experience between successive PVT and exercise-inserted PVT.

### EEG power changes in immediate effect

**Figure 4:**
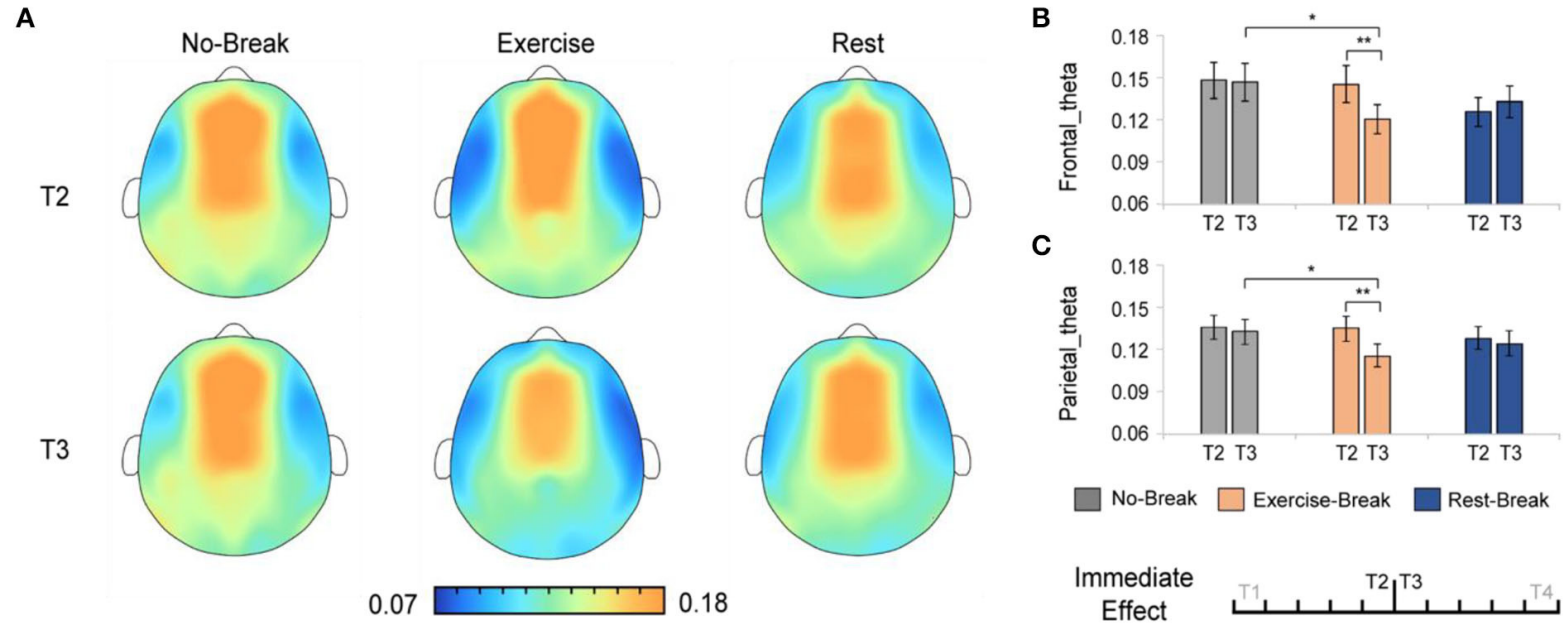
The theta spectral changes in immediate effect (T2 *vs*. T3). **(A)** The brain topography of the immediate effect of the mid-task breaks on theta activities. The EEG power ratios of the **(B)** frontal and **(C)** parietal theta in the pre-break bin (T2) and post-break bin (T3). **p < 0.01; *p < 0.05. Error bars represent the mean ± SEM.

The immediate effect of mid-task breaks on the EEG power ratio was first assessed. We observed a significant difference in theta and delta activities in T3 (post-break bin) compared with T2 (pre-break bin). Specifically, in theta band, there were significant main effects for time-by-session interaction, in P_θ_*Frontal*_ [*F*_(2, 36)_ = 5.848, *p* = 0.006^*^, η^2^ = 0.245; ^*^ indicated survive FDR threshold at *q* < 0.05 for interaction effect analysis] and P_θ_*Parietal*_ [*F*_(2, 36)_ = 3.886, *p* = 0.030^*^, η^2^ = 0.178]. Following *post-hoc* analysis indicated that, in the *Exercise-Break* session, *P*_θ_ significantly decreased in T3 compared with T2, in *P*_θ_*Frontal*_ [*F*_(1, 18)_ = 11.528, *p*= 0.003, η^2^ = 0.390], *P*_θ_*Parietal*_ [*F*_(1, 18)_ = 21.381, *p* < 0.001, η^2^ = 0.543] in comparison with a maintained *P*_θ_ in *Rest-Break* and *No-Break* sessions (Figure 4).

**Figure 5:**
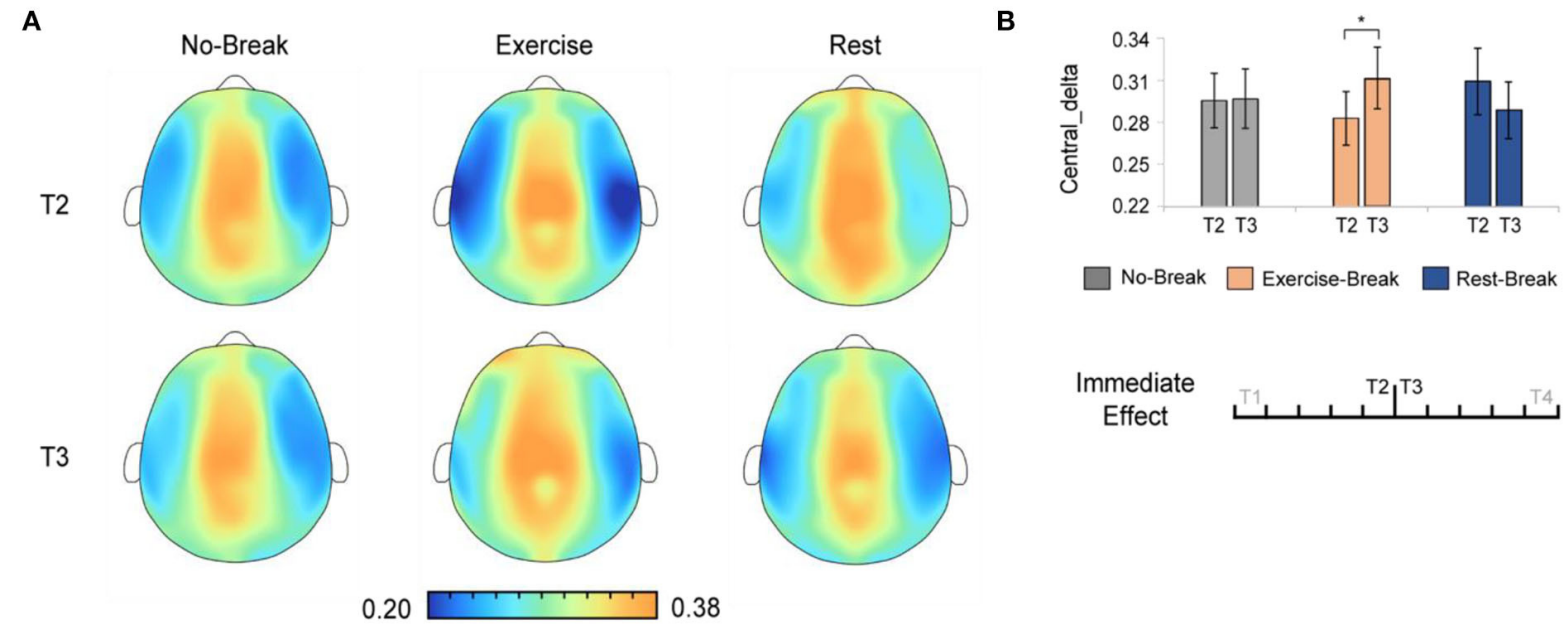
The delta spectral changes in immediate effect (T2 *vs*. T3). **(A)** The brain topography of the immediate effect of the mid-task breaks on delta activities. **(B)** The EEG power ratios of the central delta in the pre-break bin (T2) and the post-break bin (T3). *p < 0.05. Error bars represent the mean ± SEM.

And in delta band, there were significant main effects for time-by-session interaction, in *P*_δ_*Central*_ [*F*_(2, 36)_ = 3.443, *p* = 0.043, η^2^ = 0.161]. The *post-hoc* analysis indicated that, in the *Exercise-Break* session, P_δ_*Central*_ significantly increased in T3 compared with T2 [*F*_(1, 18)_ = 4.676, *p* = 0.044, η^2^ = 0.206] (Figure 5).

### EEG power changes in general effect

**Figure 6:**
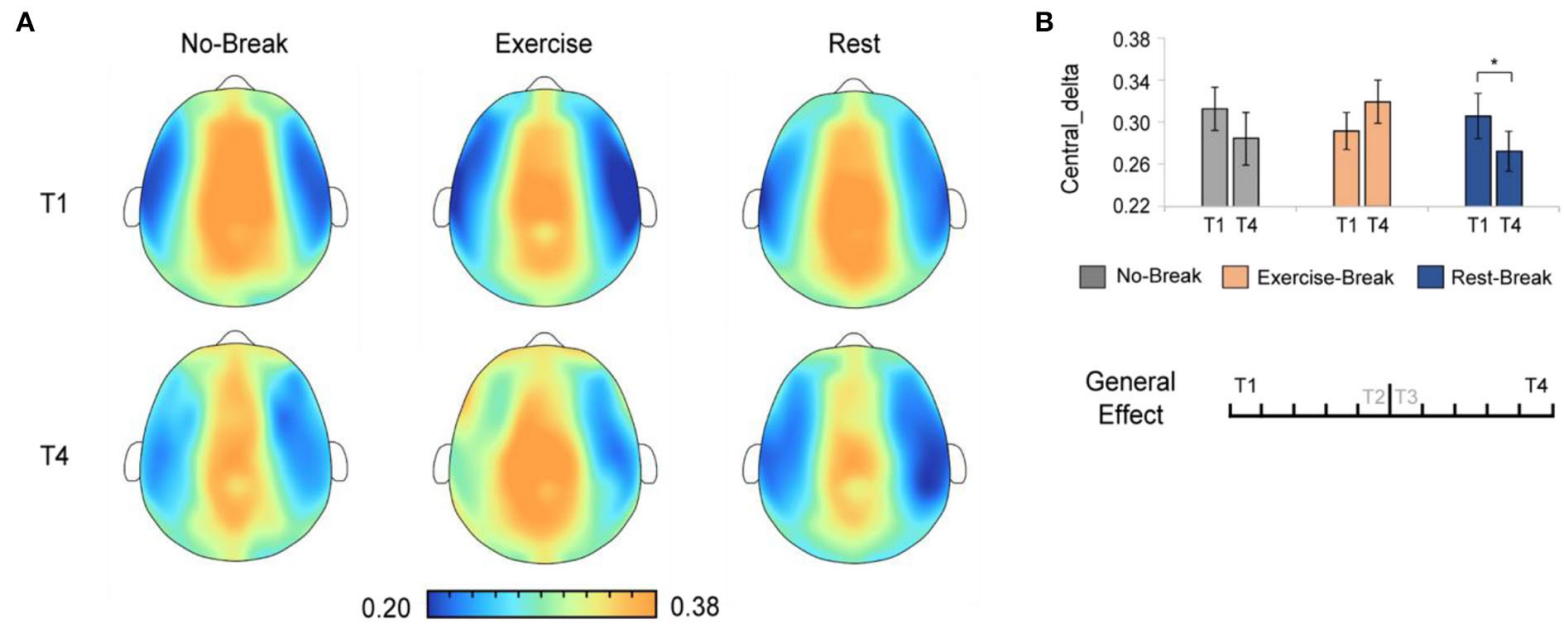
The delta spectral changes in general effect (T1 *vs*. T4). **(A)** The brain topography of the general effect of the mid-task breaks on delta activities. **(B)** The EEG power ratios of the central delta in the session-start bin (T1) and session-end bin (T4). *p < 0.05. Error bars represent the mean ± SEM.

We assessed the general effect of mid-task breaks by comparing the EEG power ratio in T4 (last 3-min bin of the whole session) with T1 (first 3-min bin). A significant main effect for time-by-session interaction was also observed in P_δ_*Central*_ [*F*_(2, 36)_ = 5.221, *p* = 0.010^*^, η^2^ = 0.225]. The *post-hoc* comparison showed a significant decrease in T4 compared to T1 [*F*_(1, 18)_ = 6.171, *p* = 0.023, η^2^ = 0.255] in the *Rest-Break* session, as well as an increase trend [*F*_(1, 18)_ = 3.212, *p* = 0.090, η^2^ = 0.151] in the *Exercise-Break* session (Figure 6).

### Correlation in EEG power and reaction time changes

**Figure 7:**
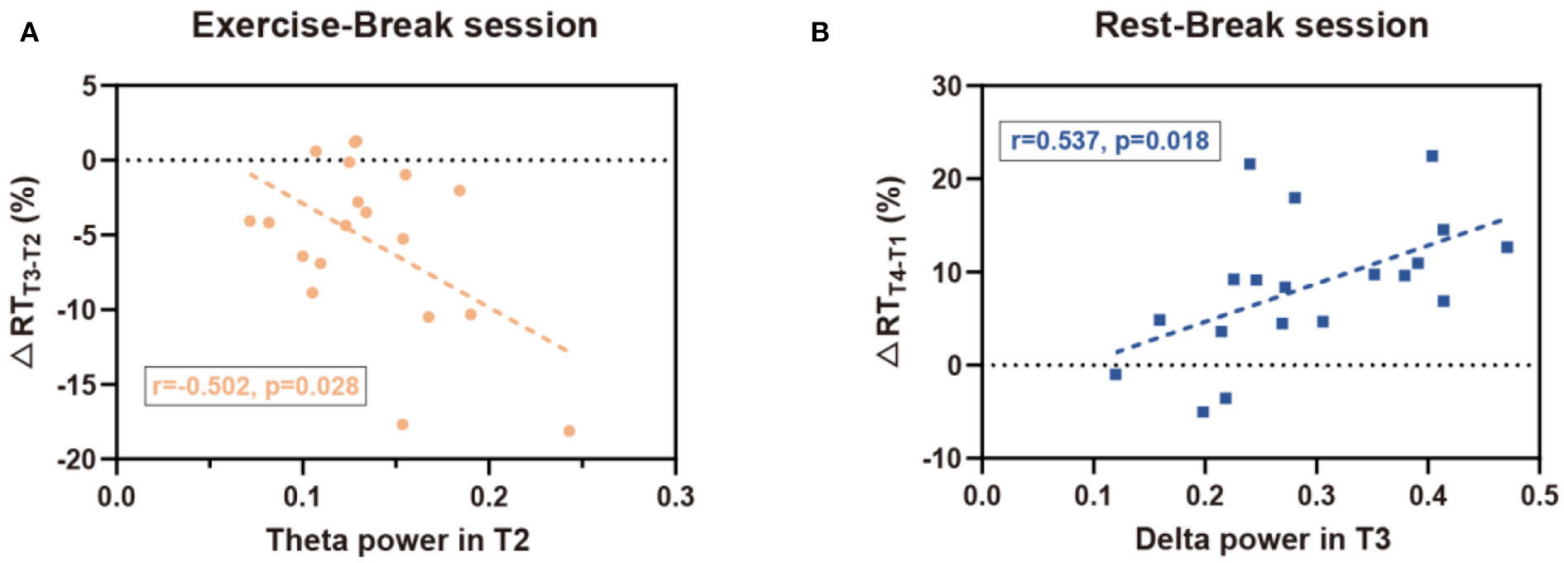
Correlation results for EEG-behavior analysis. **(A)** In the Exercise-Break session, RT change (in percentage) from T2 to T3 was negatively correlated with EEG theta power in T2. **(B)** In the Rest-Break session, RT change (in percentage) from T1 to T4 was positively correlated with EEG delta power in T3.

According to the analysis of immediate and general effect, P_δ_ and P_θ_ demonstrated significant differences immediately after the *mid-task exercise*, while P_δ_ also suggested a significant difference in the general effect of the *Rest-Break* session. Consistent with the interaction effect observed in EEG power, correlation analyses were mainly focused on the immediate effect of the *Exercise-Break* session and the general effect of the *Rest-Break* session. Correlation investigation between the EEG power and behavioral performance showed that P_θ_ in T2 was correlated with ΔRT_T3−T2_ (*r*_19_ = −0.502, *p*= 0.028) in the exercise-inserted task, and P_δ_ in T3 (*r*_19_ = 0.537, *p* = 0.018) was significantly related with ΔRT_T4−T1_ in the rest-intervention task. No significant association was observed between ΔP_θ*T*3−*T*2_ and ΔRT_T3−T2_ (*r*_19_ = 0.124, *p* = 0.613), ΔP_δ*T*3−*T*2_ and ΔRT_T3−T2_ (*r*_19_ = −0.055, *p* = 0.822) in *Exercise-Break* session, as well as ΔP_δ*T*4−*T*1_ and ΔRT_T4−T1_ (*r*_19_ = −0.425, *p* = 0.070) in *Rest-Break* session. In particular, the EEG power in correlation analysis was acquired from global electrodes rather than specific clusters to preliminarily explore the biomarker of mental fatigue recovery (Figure 7).

## Discussion

In this study, we revealed divergent post-break improvement neural patterns following mid-task exercise and rest break under similar beneficial effects on behavioral performance, and further investigated the neural basis underlying mental fatigue restoration. The significant findings are as follows: (1) behaviorally, we found a transiently restorative effect of both exercise and rest breaks on sustained attention, without any general beneficial effect on behavioral performance. (2) Exercise and rest demonstrated different patterns of EEG change in post-break improvement. In particular, theta decreased and delta increased immediately after the *mid-task exercise*, while decreased delta was induced near the end of the *Rest-Break* session. (3) Correlation analysis suggested that the immediate improvement of behavioral performance (i.e., ΔRT_T3−T2_) for exercise intervention was associated with theta power in T2. Interestingly, we found the general behavioral effect (i.e., ΔRT_T4−T1_) in the *Rest-Break* session was correlated with delta power in T3. These findings are discussed in greater detail below.

### Behavioral effects of the mid-task breaks

Both the mid-task exercise and rest induced similar immediate improvements as shown in the significantly reduced RT in the post-break window. For the *mid-task rest*, this behavioral result was inconsistent with our early findings that no significant restorative effect was observed after rest breaks (Sun et al., 2017). This discrepancy could stem from the different duration of the rest admitted to the participants, i.e., 5 min in Sun et al. (2017) *vs*. 15 min in the current work. Embracing previous observations with the current findings, we could therefore infer that a longer rest break might be needed to induce substantial recovery from mental fatigue (Ross et al., 2014; Helton and Russell, 2015). Further evidence to support this notion was from a behavioral study quantitatively investigating the effect of rest in three different durations for TOT recovery, which demonstrated that longer rest break produced a better restoration (Lim and Kwok, 2015). However, no significant recovery was observed at the end of the *Rest-Break* session in our results. This is in line with the previous study that TOT decrements tended to be steeper after long than short breaks (Lim and Kwok, 2015). As a result, providing participants with a *mid-task rest* with the time duration of 15 min might only produce a transient restoration restricted to the period right after the midway break.

The *mid-task exercise* breaks were found to be capable of replenishing the behavioral performance immediately, fairly as much as rest did. Notably, these results were consistent with accumulating evidence that aerobic exercise produced a more robust positive effect on cognitive and attention (Pontifex et al., 2019). This was also partly consistent with the arousal theory, which indicated that exercise-induced brain activation, especially in the prefrontal cortex (Popovich and Staines, 2015), the region critical for sustained attention. Our work extends the above findings by linking the positive effects of exercise to the practical application of mental fatigue restoration.

More interestingly, we found the subjective distress score in SSSQ, a measure of negative affect, kept consistent in the *Rest-Break session*, while increased in the *No-Break* session and *Exercise-Break session*. This indicated that after the 15-min cycling break, subjects might not feel recovered from mental fatigue, even though there were indeed apparent improvements in their vigilance. In addition, the trend of engagement scores in SSSQ also reflected a similar phenomenon. Put differently, although both rest and exercise could intervene TOT effect immediately, subjects might have different recognition of their “feeling about mental fatigue,” which might correspond to divergent cognitive processes underlying the similar behavioral performance improvement.

### Neural patterns following exercise-induced attention improvement

As for the *mid-task exercise*, a significant reduction of theta activity in the frontal and parietal cortex areas was observed in the immediate post-break period. Frontal theta has been repeatedly reported as a reliable index for mental fatigue (Lal and Craig, 2001; Wascher et al., 2014), and an increase in frontal theta may reflect exhausting cognitive control resources with increasing TOT (Clayton et al., 2015). For example, studies found that reduced frontal theta was associated with better performance in action execution, while higher frontal theta might reflect an excessive consumption of attention engagement and hamper the task performance (Kao et al., 2013). Besides, a significant theta power increase in the frontal was believed to reflect a more demanding attention control process that originated in the anterior cingulate cortex (ACC) (Sauseng et al., 2010). This ACC-mediated theta activity has been believed to be associated with externally oriented monitoring and executive control (Cavanagh and Frank, 2014; Cohen, 2017). From the correlation analysis, we further observed that a greater before-intervention P_θ_ was associated with more behavioral restoration after *mid-task exercise* in our study. In summary, the reductions in theta following exercise break might reflect optimized monitoring and allocation of executive control resources toward external goals so as to ameliorate TOT declines. In other words, the *mid-task exercise* matched the current attention to the desired level.

Besides, a significant increase in the central delta was also observed right after exercise intervention. Consistent with our results, the delta increase has been repeatedly reported immediately after exercise (Mechau et al., 1998; Crabbe and Dishman, 2004). The traditional view toward the increase of delta recognized it as the biomarker of fatigue. For instance, in laboratory-controlled driving simulating studies, increases in delta activity were found during the transition into the fatigued state (Lal and Craig, 2002). Unlike these findings, an increased delta ratio was observed in the current work where significant improvements in behavioral performance were revealed after cycling exercise, which might suggest a beneficial effect of the increased post-exercise delta. Of note, in addition to fatigue-related alterations, the increased delta power has also been reported to be associated with inhibition of the internal interferences that might affect the task performance (Harmony, 2013), which may lead to the observed increased post-exercise delta power. For example, during a Go/No-Go task in 15 normal young volunteers, delta activity increases were clearly demonstrated while subjects inhibited their movement (Harmony et al., 2009). Besides, another research suggested that there was a reciprocal relationship between alpha and delta activity, which reflected the inhibitory control over motivational and emotional drives (Knyazev, 2007). In summary, we inferred that the delta increases after exercise suggested an inhibitory effort toward possible interference thoughts, which in turn improved the performance and represented fatigue recovery.

In sum, these findings could fit some of the predictions in the oscillatory frequencies model of sustained attention (Clayton et al., 2015), in which attention is adjusted through the excitation of task-relevant cognitive processes and the inhibition of task-irrelevant cognitive processes. The decrease of theta after exercise could demonstrate a more effective cognitive monitoring and manipulation in primary sustained attention tasks. And the increase of delta might reflect the need for inhibiting task-irrelevant thoughts during a primary mental task. The two approaches together could promote the performance of post-break behavioral performance.

### The neural strategy of mid-task rest for handling TOT effect

In contrast with the post-exercise effect, EEG change was not observed immediately after rest intervention in low-frequency power spectral but manifested as a drop in delta power at the end of the task when the TOT effect reappeared. In addition to the biomarker of mental fatigue and internal inhibition, delta power was also considered associated with negative effects (Wu et al., 2019; Zheng et al., 2019) and subjective sleepiness (Phipps-Nelson et al., 2011). Therefore, the decrease of the delta at the end of the *Rest-Break Session* could be illustrated as the active emotion and motivation toward sustained attention task, so that participants would respond positively when mental fatigue was induced once again. This beneficial effect brought by *mid-task rest* could also be validated from the subjective feeling feedback, in which subjects maintained the effect and motivation from the start to the end of the *Rest-Break Session*, while both the *No-Break Session* and *Exercise-Break Session* suggested increased subjective distress and declined task engagement. In addition, we found that the level of delta power following rest was associated with long-term behavioral performance based on correlation analysis. In detail, a lower delta power after rest corresponded to a higher sustained attention level before the end of the task, and the delta could be used as a neurological indicator demonstrating the vigilance level after rest. In sum, *mid-task rest* improved the subjective experience to counter TOT effects, which could be manifested in the delta power after rest intervention.

### Future consideration

Some issues should be considered when interpreting our results. First, a within-subject design was adopted in this study to neutralize the widely revealed between-subject variability toward the TOT effect (Parasuraman and Jiang, 2012). Although this is an advantage of the study design, our sample size was subsequently small, and only healthy and young participants were recruited given the amounts of labor in repeated measure (i.e., each participant should come to the lab once a week for continuous 3 weeks without dropout). Therefore, more experimental studies are needed to further validate our findings with a broad age range and a larger independent sample. Besides, subjects were instructed to keep their eyes open with gaze at a central fixation cross during the mid-task rest. It is a more practical way of performing the experiment without the need to give a cue to tell subjects to open their eyes or if concern of falling asleep during the experiment that might induce additional confounding factors compared to a natural eye-closed rest. In fact, a study of multi-center neuroimaging data showed that data collection with eyes closed showed more sleep than the fixation group, suggesting that fixation supports the maintenance of wakefulness (Tagliazucchi and Laufs, 2014). Nonetheless, in a real-life situation, people intend to take a break in an ecologically valid way, with an unconstrained activity that is more relaxing. Therefore, future studies could pay more attention to the naturality and validity of the mid-task breaks to explore the practical restoration approach for a particular application scenario. Second, to induce significant performance decrement, the simple and monotonous PVT task was introduced in this study, which was considered to be more stressful and effortful than complex tasks (Langner and Eickhoff, 2013). In fact, handling multiple tasks were common and important in daily life, with a series of decision-making, alertness, or physical movement, corresponding to more sophisticated activating states of the brain. Therefore, future work could consider the paradigm design consisting of simulated real-world working assignments to further verify the effectiveness of the divergent fatigue-intervention strategy. Third, based on the cortical oscillation model (Clayton et al., 2015), we found that the excitation of task-relevant cognitive processes and the inhibition of task-irrelevant processes could explain the EEG change in theta and delta following *mid-task exercise*. It is noteworthy mentioning that we did not directly distinguish the task-irrelevant cognitive state from the performance of external stimuli response in the current work. As a result, future research may especially focus on the brain status after break intervention through the neurofeedback technique to further investigate our novel interpretation.

## Conclusion

In summary, this study provided one of the first looks into the neural mechanism of recovery strategy recruited by different breaks, through evaluating the behavioral performance and spatio-spectral characteristics of low-band EEG following rest and exercise break in a sustained attention task. Our findings provided behavioral and electrophysiological evidence to demonstrate that divergent neural patterns were recruited by mid-task rest and exercise though vigilance restoration was similar, indicating different neural strategies to counter TOT effects. Our work broadens the knowledge of mental fatigue countermeasure and may lead to further mechanism study in neuroscience and psychology, as well as real-world ergonomics for various TOT restoration situations.

## Data availability statement

The raw data supporting the conclusion of this article is available upon reasonable request to the corresponding authors.

## Ethics statement

The studies involving human participants were reviewed and approved by Zhejiang University. The patients/participants provided their written informed consent to participate in this study.

## Author contributions

Conceptualization, resources, and funding acquisition: PQ and YS. Methodology: SW, LZ, LG, JY, GL, PQ, and YS. Formal analysis: SW and LZ. Writing—original draft preparation: SW, LZ, and YS. Supervision: YS. All authors have read and agreed to the published version of the manuscript.

## Funding

This work was funded by the National Natural Science Foundation of China (Grant Nos. 82172056, 51905379, and 81801785), the National Key Research and Development Program of China (Grant No. 2021ZD0200400), the Key Research and Development Program of Zhejiang Province (Grant No. 2022C03064), the Zhejiang University Global Partnership Fund (Grant No. 100000-11320), and the Hundred-Talent Program of Zhejiang University. This work was partially supported by the Shanghai Municipal Science and Technology Major Project (Grant No. 2021SHZDZX0100) and the Fundamental Research Funds for the Central Universities.

## Conflict of interest

The authors declare that the research was conducted in the absence of any commercial or financial relationships that could be construed as a potential conflict of interest.

## Publisher's note

All claims expressed in this article are solely those of the authors and do not necessarily represent those of their affiliated organizations, or those of the publisher, the editors and the reviewers. Any product that may be evaluated in this article, or claim that may be made by its manufacturer, is not guaranteed or endorsed by the publisher.
